# Cardiac monocytes and macrophages after myocardial infarction

**DOI:** 10.1093/cvr/cvz336

**Published:** 2019-12-16

**Authors:** Claire Peet, Aleksandar Ivetic, Daniel I Bromage, Ajay M Shah

**Affiliations:** School of Cardiovascular Medicine and Sciences, James Black Centre, King's College London BHF Centre of Excellence, 125 Coldharbour Lane, London SE5 9NU, UK

**Keywords:** Monocyte, Macrophage, Myocardial infarction

## Abstract

Improvements in early interventions after acute myocardial infarction (AMI), notably, the increased use of timely reperfusion therapy, have increased survival dramatically in recent decades. Despite this, maladaptive ventricular remodelling and subsequent heart failure (HF) following AMI remain a significant clinical challenge, particularly because several pre-clinical strategies to attenuate remodelling have failed to translate into clinical practice. Monocytes and macrophages, pleiotropic cells of the innate immune system, are integral in both the initial inflammatory response to injury and subsequent wound healing in many tissues, including the heart. However, maladaptive immune cell behaviour contributes to ventricular remodelling in mouse models, prompting experimental efforts to modulate the immune response to prevent the development of HF. Seminal work in macrophage biology defined macrophages as monocyte-derived cells that are comprised of two populations, pro-inflammatory M1 macrophages and reparative M2 macrophages, and initial investigations into cardiac macrophage populations following AMI suggested they aligned well to this model. However, more recent data, in the heart and other tissues, demonstrate remarkable heterogeneity and plasticity in macrophage development, phenotype, and function. These recent insights into macrophage biology may explain the failure of non-specific immunosuppressive strategies and offer novel opportunities for therapeutic targeting to prevent HF following AMI. Here, we summarize the traditional monocyte-macrophage paradigm, experimental evidence for the significance of these cells in HF after AMI, and the potential relevance of emerging evidence that refutes canonical models of monocyte and macrophage biology.

## Graphical Abstract

**Graphical Abstract:**
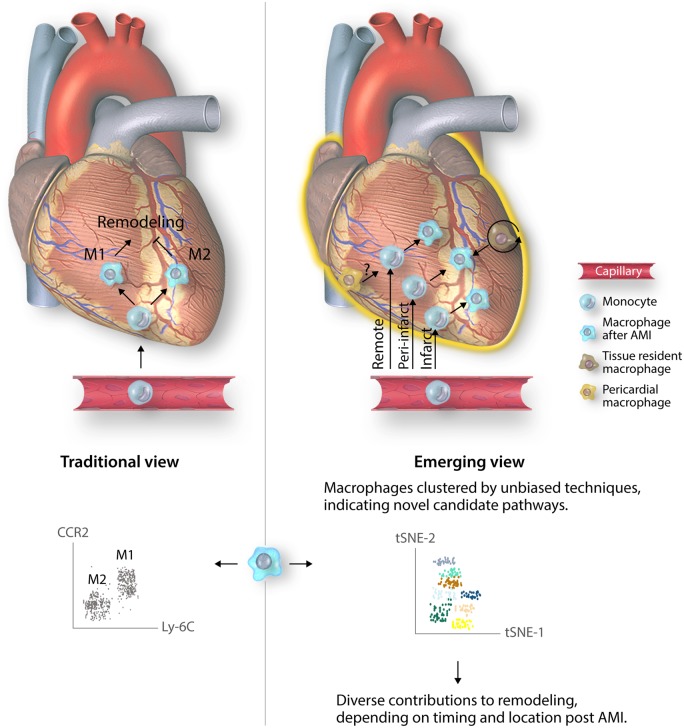

## 1. Introduction

Emergency management of acute myocardial infarction (AMI) has been revolutionized by timely reperfusion therapy and, in particular, increasing access to primary percutaneous coronary intervention, leading to dramatic improvements in early survival.^1–3^ Despite this, AMI remains the commonest cause of heart failure (HF) and HF-related morbidity and mortality following AMI remain high.^4–7^ In view of the burden of HF following AMI, there is an unmet need for better understanding of the pathogenesis of ventricular remodelling and development of novel therapeutic targets.

In AMI, reduced blood flow to a region of myocardium results in infarction, mediated mainly through oncosis and necrosis.^8^ This necrotic myocardium becomes a region of mechanical weakness that requires scar deposition to prevent myocardial rupture and limit functional deterioration. This adaptive remodelling is necessary to prevent early mortality following AMI. However, excessive and progressive (maladaptive) ventricular remodelling, at both the infarct site and remote myocardium, alters ventricular size and function, culminating in the clinical syndrome of HF. Understanding the balance between adaptive and maladaptive remodelling is an emerging research area that has the potential to identify novel therapeutic targets for the prevention of HF after myocardial infarction (MI).

The innate immune response is an important regulator of this process and comprises three key phases: the inflammatory, proliferative, and maturation phases.^8–10^ Cell death triggers sterile inflammation, through exposure of endogenous damage-associated molecular patterns (DAMPs) to the innate immune system. Recognition of DAMPs by pattern recognition receptors on resident innate immune cells prompts a cascade of chemokine and pro-inflammatory cytokine release, recruiting and activating neutrophils, monocytes, and macrophages. Together, these cells degrade the extracellular matrix and phagocytose necrotic cells. This initial inflammatory phase, which peaks around Day 3 post-injury, is followed by a longer proliferative phase lasting around 10 days, also mediated by immune cells. This phase is characterized by anti-inflammatory signalling, fibroblast proliferation, and deposition of granulation tissue. The final, maturation phase occurs over subsequent months and is characterized by remodelling of the extracellular matrix, with few immune cells present at the site of injury. During this period following AMI, there are significant changes to ventricular size, shape, and function, with maladaptive remodelling leading to the development of HF.^11^

Monocytes and macrophages are implicated at all three stages of this response. Cardiac monocyte and macrophage numbers expand rapidly in the days following AMI.^12^^,^^13^ These initial, infiltrating populations demonstrate a pro-inflammatory phenotype which shifts over the ensuing days to a predominantly reparative phenotype, coordinating the deposition of scar tissue.^14^ By 2 weeks post-AMI, monocyte and macrophage populations at the site of infarction return to baseline, although macrophages persist for months after AMI in the remote, remodelling myocardium.^15^

Nonetheless, numerous trials of immunosuppressive agents have failed to mitigate HF following AMI, which is unsurprising given the complex interplay of reparative and deleterious processes, and demonstrates the need for a more detailed understanding of cellular and molecular pathways in ventricular remodelling to facilitate more targeted therapeutic interventions.^16–18^

The putative role of monocytes and macrophages in healing post-AMI, and the persistence of macrophages in remodelling ventricular myocardium, has led to interest in targeting these cells to prevent maladaptive remodelling. However, various strategies to target specific sub-populations have delivered seemingly contradictory results. This may relate to an overly constrictive traditional understanding and definitions of macrophage subsets, which are increasingly understood to be heterogeneous and plastic, impeding focused investigation of their role in ventricular remodelling after AMI.

Here, we discuss the canonical view of monocyte-derived macrophage differentiation and activation, and how strategies targeting monocyte and macrophage populations have impacted on ventricular remodelling in pre-clinical models. We then discuss emerging data on the complexity of macrophage development and phenotype that challenges the existing paradigm and may justify disparities in existing evidence. Finally, we discuss how recognition of macrophage heterogeneity and plasticity may benefit ongoing investigation and the development of new treatments in this important clinical area.

## 2. Traditional views of cardiac monocyte and macrophage populations

### 2.1 Monocyte and macrophage classification

The mononuclear phagocyte system is a family of myeloid cells comprising monocytes, macrophages, and dendritic cells. Monocytes are short-lived circulating cells that are implicated in inflammation through both direct effects and by differentiation into dendritic cells and macrophages. These cells develop from the common myeloid progenitor in the bone marrow and are released into the circulation, where they comprise two main subsets: conventionally known as classical and non-classical. These populations were originally described in humans, defined by CD16 (cluster of differentiation 16) expression, and later in mice. Phenotypic profiling suggests that these populations correspond, facilitating experimental characterization of populations in human studies.^19^^,^^20^

Classical monocytes, typically described as CD14^+^CD16^neg^ in humans and Ly-6C^high^ in mice, are implicated in inflammation.^21^^,^^22^ These cells are released from the bone marrow and extramedullary sites of haematopoiesis, such as the spleen, and traffic to sites of injury in a CCR2 [chemokine (C-C motif) receptor 2] dependent manner, and comprise over 90% of circulating monocytes.^22^ Upon extravasation, classical monocytes contribute to the innate immune response both via direct effects, such as tumour necrosis factor α (TNF-α) and nitric oxide production, and indirectly, by differentiation into macrophages and dendritic cells.^23^ More recently, these cells have been shown to play an important role in activating the adaptive immune response, through antigen presentation to T cells.^23^

In contrast, non-classical monocytes (CD14^+^CD16^+^/Ly-6C^low^) are thought to persist in the circulation and their role in inflammation is less clear. In mice, these cells have been demonstrated to arise in an NR4A1 (nuclear receptor subfamily four group A member one) dependent manner, a transcription factor which is dispensable for classical monocyte development.^24^^,^^25^ Both murine and human non-classical monocytes may be differentiated from classical monocytes by high levels of CX_3_CR1 (C-X3-C motif chemokine receptor 1) expression and, in mice, this receptor has been demonstrated to be essential for their survival.^22^^,^^26^^,^^27^ These cells patrol the endothelium and may play a role in immune surveillance.^28^^,^^29^

On entry into tissues, monocytes give rise to dendritic cells and macrophages. Macrophages phenotypically differ from monocytes by increased expression of CD68 and MHCII (major histocompatibility 2), as well as F4/80 in mice, and reduced expression of CD14 in humans. Originally described as the prototypic phagocytic cells, macrophages are now recognized to mediate incredibly diverse processes, from cytokine production, phagocytosis, and co-ordination of the formation of granulation tissue in the context of inflammation, to organ-specific homoeostatic functions, the scope of which is still emerging in the heart.

Investigation of macrophage phenotype *in vitro* led to the widespread use of the M1 and M2 polarization model to describe macrophages in the context of inflammation.^30^ Macrophages stimulated with pro-inflammatory signals, such as LPS (lipopolysaccharide) and IFN-γ (interferon-gamma), demonstrate a stereotypic pro-inflammatory transcriptome and behaviour. These M1, or classically activated, macrophages demonstrate enhanced phagocytosis, antigen presentation on MHC II, and generation of reactive oxygen species. They also produce and release pro-inflammatory cytokines, such as IL-12 (interleukin 12), IL-23, IL-27, and TNF-α; chemokines, including CXCL9 (CXC motif chemokine ligand 9), CXCL10, CXCL11; and matrix metalloproteinases (MMP-1, 2, 7, 9, 12). Together these contribute to a pro-inflammatory micro-environment.

In contrast, macrophages stimulated with anti-inflammatory cytokines, such as IL-4 and IL-13, show expression of characteristic anti-inflammatory genes and a reparative phenotype.^31^ M2, or alternatively activated, macrophages produce anti-inflammatory cytokines (IL-10); chemokines, including CCL17 (C-C motif chemokine ligand 17),^22^^,^^24^ and growth factors, such as vascular endothelial growth factor and tumour growth factor beta. Together these mediators stimulate fibroblast-mediated production of the extracellular matrix, cell proliferation and angiogenesis, promoting tissue remodelling and repair.

Following these seminal studies, the M1–M2 activation paradigm continues to be widely used.

### 2.2 Cardiac monocyte and macrophage populations in health and disease

During homoeostasis, both classical and non-classical monocytes are found in the coronary vasculature. Intravital microscopy demonstrates classical monocytes circulate rapidly, whereas non-classical monocytes circulate more slowly, crawling along the endothelium.^12^^,^^28^^,^^29^ These patrolling monocytes appear to play in role in immuno-surveillance. Within the myocardium, macrophages are present in both the murine and human heart, comprising 6–8% of non-cardiomyocytes in the adult mouse.^32^^,^^33^ These cardiac macrophages play a role in cardiac development, immuno-surveillance and may contribute important specialized cardiac functions, such as conduction, though their exact functional significance is still emerging.^10^

Following AMI, monocyte and macrophage populations expand at the site of infarction and change their phenotype dramatically in the murine heart. Intravital microscopy demonstrates that monocyte recruitment to the infarct begins as early as 30 min following AMI, first from the vascular pool and later from the splenic reservoir.^12^ This initial recruitment is rapidly overtaken by neutrophil infiltration, and recent evidence suggests these sentinel infiltrating monocytes may play a role in neutrophil attraction.^34^

The infiltration of monocyte and macrophage populations into infarcted tissue occurs in two sequential phases.^13^^,^^14^ Ly-6C^high^ monocytes are the predominant population early post-AMI, peaking between Days 3 and 5, and demonstrating a pro-inflammatory phenotype, including TNFα expression and high proteinase activity. Ly-6C^high^ macrophages form the principal macrophage subset during this early phase, though they are less abundant than monocytes.^35^^ ^As inflammation resolves, Ly-6C^low^ cells predominate, peaking in the infarct on Day 7 post-AMI.^14^ Initially described as a distinct wave of Ly-6C^low^ monocyte infiltration, more refined gating strategies and lineage tracing experiments have demonstrated these cells to be principally Ly-6C^low^ macrophages, which are derived from Ly-6C^high^ monocytes.^35^

**Figure 1:**
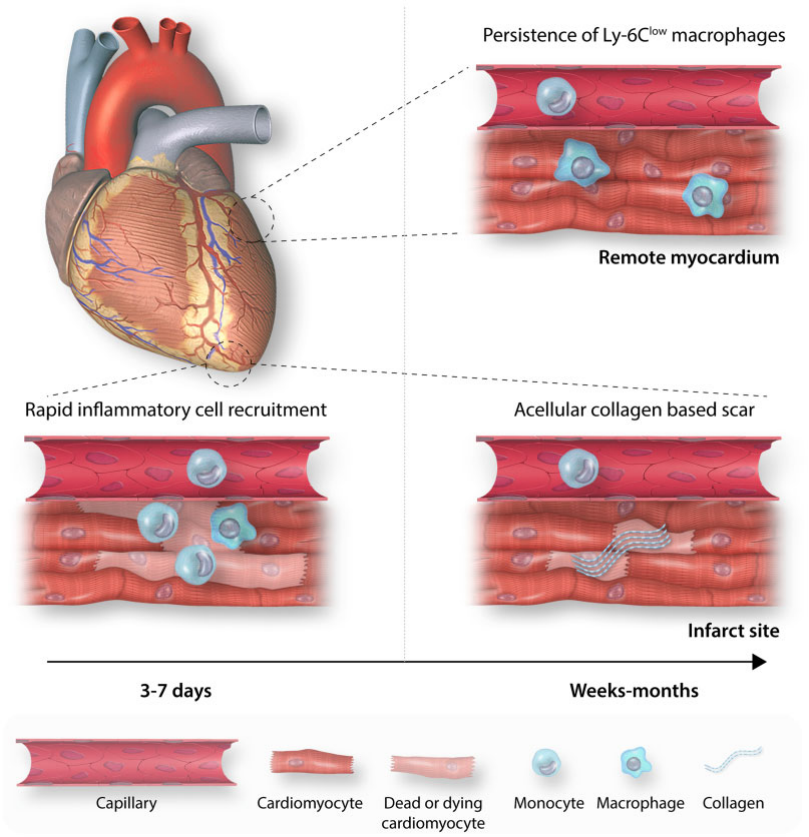
Myocardial monocyte and macrophage populations after AMI. (*A*) Infarct site inflammatory cell populations expand rapidly following AMI with an initial expansion of Ly-6C^high^ monocytes, peaking Day 3–5, followed by an expansion of Ly-6C^low^ macrophages, peaking on Day 7. (*B*) By 6 weeks, inflammatory cell infiltration at the infarct site has returned to baseline, leaving an acellular collagen based scar, whereas Ly-6C^low^ macrophages persist in the remote, remodelling myocardium.

A similar pattern of sequential Ly-6C^high^ and Ly-6C^low^ expansion is observed in the remote myocardium.^13^ However, Ly-6C^low^ numbers peak 5 days later than at the infarct site. Importantly, in contrast to the site of ischaemia, macrophages and Ly-6C^high^ monocytes persist in the non-ischaemic myocardium (*Figure 1*).^15^

These changes in monocyte and macrophage populations in the myocardium following AMI are accompanied by an expansion of circulating monocytes.^14^^,^^36^ The major source of circulating monocytes following AMI is the splenic reservoir, and these cells are generated both by seeding from the bone marrow and by local monocytopoiesis.^36–38^ The spleen contains myeloid precursors that expand rapidly after AMI; on adoptive transfer of splenic granulocyte-macrophage progenitors after AMI, these cells differentiate into splenic macrophages that mobilize to the healing myocardium.^38^ Monocytopoiesis at both medullary and extramedullary sites is regulated by sympathetic nervous signalling and IL-1, both in the acute and chronic phase following AMI.^15^^,^^38^^,^^39^

Human studies of cardiac populations following AMI demonstrate a similar pattern of infiltration to that observed in mice. Humans demonstrate peripheral blood monocytosis after AMI, with sequential peaks of CD14^+^CD16^neg^ and CD14^+^CD16^+^ monocytes on Days 3 and 5, respectively, though CD14^+^CD16^neg^ monocytes are more abundant throughout.^40^ This was confirmed in an analysis of post-mortem cardiac tissue, which showed a predominance of CD16^−^ cells during the inflammatory phase, compared to comparable populations of CD16^+^ and CD16^−^ cells during the proliferative phase.^41^ Both the bone marrow and splenic reservoir of monocytes (defined as CD14^+^ cells) were significantly depleted in this study.

## 3. Monocyte–macrophage phenotype and ventricular remodelling

### 3.1 Pro-inflammatory phenotypes correlate with ventricular dysfunction in mice and humans

The role of monocytes and macrophages in wound healing and the observation of two distinct waves of infiltration after AMI has led to the hypothesis that these cells may have a role in maladaptive ventricular remodelling and the development of HF. Observational studies in mice and humans demonstrate a correlation between classical monocytosis and degree of LV dysfunction after AMI. For example, the *ApoE^neg/neg^* (Apoprotein E) model demonstrates a chronically expanded pool of circulating Ly-6C^high^ monocytes.^42^ Following coronary artery ligation, these mice show increased myocardial infiltration and persistence of Ly-6C^high^ monocytes on Day 5 and reduced left ventricular ejection fraction (LVEF) at 3 weeks, compared to wild-type mice. Similarly, in patients following ST-segment elevation MI (STEMI), there is a negative correlation between peripheral blood monocytosis and recovery of ventricular function.^43^ In one study, peak circulating CD14^+^CD16^neg^ levels following STEMI negatively correlated both with wound healing (assessed by the extent of myocardial salvage on cardiac magnetic resonance imaging at Day 7) and LVEF at 6 months, though the degree of HF at follow-up is likely to have been confounded by the greater initial infarct size.^40^

These findings have been corroborated when classical monocytes are defined as CD14^+^CD62L^+^ cells, with high levels associated with greater infarct size and regional systolic LV dysfunction at 4-month follow-up after STEMI, which remained significant even after stratification according to the extent of transmural infarction.^44^ An inverse trend was observed with the number of non-classical monocytes (CD14^+^CD62L^neg^), with high levels correlating to improved LV function, though this was not statistically significant. However, crucially, these patients were taken from the HEBE trial, where subjects received intracoronary infusions of autologous peripheral blood mononuclear cells (PBMC) following AMI. Given the expansion of circulating classical monocyte populations following AMI, the outcomes at later time points are likely to be confounded by the intracoronary infusion of these pro-inflammatory skewed PBMC populations. The trial itself failed to show a significant effect of intracoronary infusion of autologous PBMC on left ventricular size or function at 4-month follow-up and, interestingly, revealed increased mortality and a reduction in recovery of left ventricular function at 24 months when compared with control.^45^^,^^46^

### 3.2 Targeting circulating monocytes and their reservoirs

**Figure 2:**
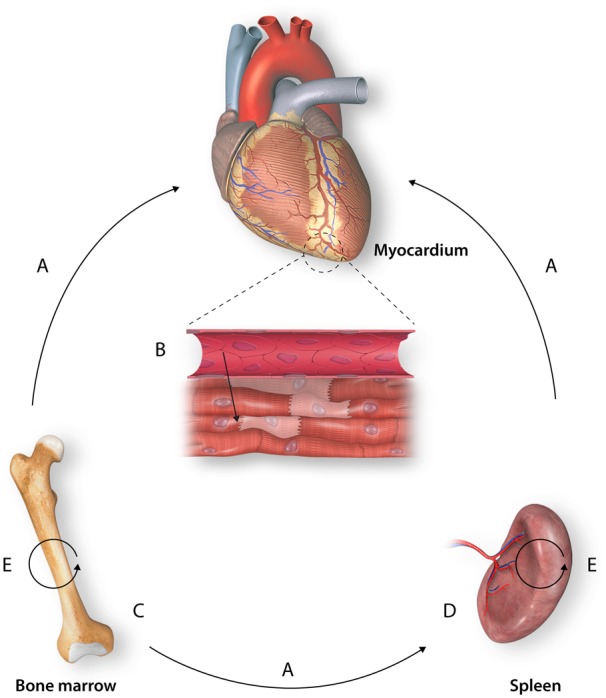
Targeting circulating monocytes and their reservoirs. Approaches depleting myocardial monocyte populations following AMI have focused on various stages of their trafficking upstream. (*A*) Depletion of all circulating monocytes (e.g. clodronate-loaded liposomes). (*B*) Inhibition of monocyte entry into the myocardium (e.g. *Ccr2^neg/neg^*). (*C*) Inhibition of monocyte release from the bone marrow (e.g. *Ccr2^neg/neg^*). (*D*) Depletion of the splenic reservoir or inhibition of splenic release (e.g. splenectomy, Agt1ar antagonism). (*E*) Inhibition of monocytopoiesis (e.g. beta-adrenergic blockade).

To better understand, the specific contributions of monocyte and macrophage subsets on remodelling following AMI, studies have focused on either blocking monocyte recruitment to the myocardium or manipulating the phenotype of myocardial monocytes and macrophages (*Figure 2*).

The correlation of monocytosis with poor outcomes led to the hypothesis that excessive monocyte infiltration is deleterious. However, treatment with clodronate-loaded liposomes after AMI, which deplete circulating monocytes (*Figure 2A*), either during the early pro-inflammatory phase or later proliferative phase, leads to impaired infarct repair.^14^ In both instances, reduced collagen deposition and angiogenesis are seen during the proliferative phase, whilst only depletion of early monocytes increases necrotic cells and debris, suggesting that both the pro-inflammatory early phase and later reparative phase are essential for infarct healing.

A number of studies have, therefore, sought to selectively target trafficking of Ly-6C^high^ monocytes to the myocardium, taking advantage of the observation that Ly-6C^low^ monocytes traffic independently of CCR2 (*Figure 2B*). Following coronary artery ligation, *Ccr2^neg/neg^*mice demonstrate reduced myocardial infiltration of Ly-6C^high^ cells and siRNA knockdown of *Ccr2* in wild-type mice reduces Ly-6C^high^ monocyte infiltration of the myocardium and improves LVEF and left ventricular end-diastolic volume (LVEDV) in these mice.^14^^,^^47^ Notably, CCR2 is not only important for attraction of Ly-6C^high^ monocytes to the myocardium but is also necessary for their mobilization from the bone marrow, and therefore, these models are likely to be exerting some of their effect via bone marrow mobilization and, in the case of constitutive knockout, prior seeding of the spleen by monocytes that originated in the bone marrow (*Figure 2C*).^48^

However, while targeting CCR2 can impede Ly-6C^high^ monocyte trafficking, release of monocytes from the spleen occurs independently of CCR2 and splenic monocytes have been demonstrated to contribute to maladaptive ventricular remodelling (*Figure 2D*).^36^ Splenectomy in mice 8 weeks post-coronary artery ligation appears to improve ventricular function, as assessed by LVEDV and LVEF, whilst control mice that received sham abdominal surgery demonstrated ongoing deterioration of LV function.^37^ Similarly, adoptive transfer of splenic monocytes from mice with ventricular dysfunction post-AMI caused maladaptive ventricular remodelling in the recipients. In contrast, adoptive transfer of LPS-stimulated splenic monocytes did not induce ventricular remodelling, suggesting alterations specific to the splenic niche in the context of HF modulate monocyte phenotype, and that this phenotype is functionally distinct from that of *in vitro* derived pro-inflammatory monocytes. Interestingly, release of splenic monocytes has been shown to depend on the angiotensin II Type 1a receptor, and knockout of this receptor reduces both circulating and myocardial monocytes following AMI.^36^ It is possible that the effect of drugs that inhibit the renin–angiotensin system, which are in routine use in the prevention and treatment of HF following AMI, is partially mediated via this mechanism.

A final target to deplete myocardial monocyte populations is monocytopoiesis (*Figure 2E*). Targeting IL-1β, a cytokine that promotes monocytopoiesis following AMI, reduces circulating monocyte levels following coronary artery ligation and mitigated deterioration of LV function at 3 weeks.^49^ These findings are pertinent in light of the recent success of the monoclonal antibody to IL-1β, canakinumab, in prevention of cardiovascular events in patients with previous AMI.^50^ Sympathetic nervous system signalling has been demonstrated to drive monocytopoiesis and the routine use of β adrenergic blockade as cardio-protection following AMI may be exerting some effect via this mechanism.^15^ The impact on myocardial monocyte infiltration after AMI of inhibitors of both the renin–angiotensin system, implicated in splenocyte release, and the β adrenergic system, implicated in monocytopoiesis, remains to be characterized. Without such characterization, efforts to translate putative strategies targeting these cells may be confounded by the unknown impact of these commonly prescribed medications.

Other studies have hypothesized that Ly-6C^low^ monocytes may protect against ventricular remodelling by attenuating inflammation. Mice deficient for *Nr4a1*, an obligate transcription factor for Ly-6C^low^ monocyte development, showed absent circulating and myocardial Ly-6C^low^ monocytes and impaired LV function, with reduced LVEF and increased LVEDV at 21 days post-coronary artery ligation, compared to wild-type mice.^35^ However, rather than mediating its effects solely through depletion of Ly-6C^low^ monocytes, the authors demonstrated that *Nr4a1* deletion also modulated the behaviours of Ly-6C^high^ monocytes and both macrophage subsets. In particular, Ly-6C^high^ monocytes demonstrated increased expression of CCR2 and infiltration of the infarct site was increased. Absolute numbers of Ly-6C^high^ and Ly-6C^low^ macrophages were increased in the myocardium on Day 7, and global macrophage transcription was skewed towards a pro-inflammatory phenotype, with increased expression of IL-1βα, TNFα, and IL-6, and reduced expression of canonical M2 marker, CD206.

### 3.3 Targeting myocardial macrophage phenotype

NR4A1 is one of a number of identified molecular targets that links macrophage phenotype with ventricular remodelling. Initial studies showed that up-regulating canonical M2 genes in myocardial macrophage populations, using IL-4 or IL-10 treatment or GABA_A_R (gamma aminobutyric acid A receptor) agonism, following coronary artery ligation protects against ventricular remodelling.^51–53^ However, these approaches are non-specific as they also manipulate other cell types, including T-reg cells, and fail to demonstrate a causal role for macrophages or any particular subset.

Specific targeting of macrophage polarization, therefore, provides more convincing evidence of a causative role for macrophages in both early infarct repair and ventricular remodelling. Knockout of *Trib1* (Tribbles pseudokinase 1) causes a selective depletion of CD206^+^ macrophages, a population described in this study as M2 macrophages.^53^ Following coronary artery ligation, *Trib1* deficient mice demonstrate a nine-fold increase in cardiac rupture, and this phenotype is salvaged by adoptive transfer of CD206^+^ macrophages, providing evidence of a beneficial role for reparative macrophages early in the inflammatory phase. *Trib1^neg/neg^* demonstrated impaired LV function compared to wild type, implicating this macrophage population in protecting against ventricular remodelling. Similarly, in an *Mmp28* knockout model, which shows reduced capacity of peritoneal macrophages to differentiate into M2 macrophages *in vitro*, ventricular function was impaired at 7 days compared to wild types after coronary artery ligation.^54^ Cardiac macrophage numbers were not significantly altered, but expression of canonical M1 markers was increased and M2 markers were reduced.

Efferocytosis, the phagocytosis of apoptotic cells, has been hypothesized as a cell-intrinsic mechanism by which macrophages might shift from a pro-inflammatory to reparative phenotype. Constitutive knockout of *Mertk* (Mer tyrosine protein kinase), a receptor tyrosine kinase required for effective efferocytosis, led to increased expression of canonical M1 genes in macrophages and exacerbated deterioration in LV function after coronary artery ligation.^55^

Conversely, blocking pathways implicated in M1 macrophage polarization was hypothesized to protect cardiac function after coronary artery ligation. siRNA targeting of *Irf5* (interferon regulatory factor 5), a transcription factor implicated in M1 polarization, resulted in reduced expression of the canonical M1 markers TNF-α and IL-1β, but no change in M2 marker expression on Day 4 post-coronary artery ligation.^56^ These mice had similar infarct sizes but knockdown reduced LV dilatation at 3 weeks.

These observations led to the hypothesis that shifting the macrophage phenotype from the pro-inflammatory M1-like macrophage to an anti-inflammatory M2-like macrophage protects the heart from adverse remodelling following AMI. However, more recent evidence has challenged the use of the M1–M2 paradigm by demonstrating improved outcomes following knockout of *Gata3* (GATA-binding factor-3), a transcription factor implicated in the differentiation of M2 macrophages.^57^ Selective knockout of *Gata3* in myeloid cells depleted Ly-6C^low^ but not Ly-6C^high^ macrophages following AMI and, surprisingly, the Ly-6C^low^ macrophage depleted mice demonstrated improved LV size and function at 2 months post-AMI. Additionally, absolute numbers of Ly-6C^high^ macrophages increased in the knockout model, challenging the view that the presence of excessive early pro-inflammatory populations is deleterious.

To reconcile this study with the view that M2 macrophages are protective requires careful re-examination of the canonical M1–M2 macrophage model. The variety and limitations of methods to define macrophage populations means that cell populations described in various studies may not necessarily align to one another. Emerging analyses of the wide phenotypic spectrum of macrophages, as well as the existence of non-monocyte-derived macrophage subsets, supports this view.

## 4. Insights into the complexity of mononuclear cell populations following AMI

### 4.1 Macrophages *in vitro* and *in vivo* exhibit a network of stimulus-dependent phenotypes

The M1–M2 paradigm is based on the polarization of bone marrow-derived macrophages in response to LPS and IFN-γ or IL-4 and IL-13.^30^ However, when human macrophages are stimulated *in vitro* with a more diverse range of cytokines than originally described, many more distinct phenotypes result, with one study eliciting 299 distinct transcriptomes that do not map along a spectrum from M1 to M2.^58^ Similarly, the co-expression of canonical M1 and M2 markers in both mouse and human macrophages is well characterized, as is the ability of M1 or M2 macrophages to acquire canonical markers of the other subset *in vitro.*^59^^,^^60^ Accordingly, the M1–M2 paradigm is widely accepted as an over-simplification, and yet it continues to guide experimental study design *in vivo*.^61^

**Figure 3:**
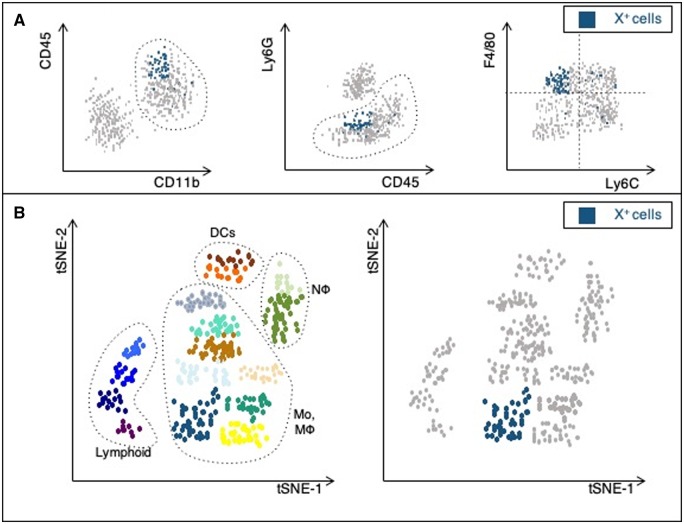
Advantages of high throughput techniques to examine immune cell heterogeneity. (*A*) Flow cytometry is widely used to immunophenotype monocytes and macrophages. A common gating strategy in mice defines these cells as CD45^+^CD11b^+^Ly-6G^low^. Based on Ly-6C and F4/80 expression these cells are commonly described as classical (Ly-6C^high^F4/80^low^) and non-classical monocytes (Ly-6C^low^F4/80^low^), and M1 ((Ly-6C^high^F4/80^high^) and M2 (Ly-6C^low^F4/80^high^). Cells expressing a particular transcription factor, termed X, may not align well with the M1/M2 paradigm, Knockout of this transcription factor may preferentially deplete a particular population, such as M2 macrophages, but is neither sensitive nor specific, failing to deplete all ‘M2’ macrophages, and also depleting cells of other subsets. (*B*) Novel high throughput techniques allow unbiased resolution of cells into distinct populations, based on gene expression profile (scRNA-seq) or large panels of differentially expressed proteins (mass cytometry). Such methods allow identification of candidate pathways for therapeutic targeting and ensure knockout models selectively deplete the population of interest. DC, dendritic cells; Mo, monocytes; Mϕ, macrophages; Nϕ, neutrophils.

With this in mind, studies targeting particular transcriptional pathways are likely to be affecting only a subpopulation of M1 or M2 cells. For example, knockout of *Gata3* depletes CCR2^neg^Ly-6C^low^ macrophages but does not eliminate them, suggesting that only a proportion of these cells are *Gata3*-dependent (*Figure 3A*). It is, therefore, misleading to conclude that M2 macrophages are responsible for the observed experimental phenotype. This also demonstrates the limitations in current definitions of macrophage subsets, as markers used to describe M1 and M2 populations vary between studies, and accordingly, the populations described are not comparable.

Novel techniques that allow resolution of gene expression at the single-cell level, including single-cell RNA sequencing (scRNA-seq) and mass cytometry, may help overcome these issues (*Figure 3B*). scRNA-seq assesses the transcriptome at single-cell level and enables unbiased clustering of cells based on common expression profiles. scRNA-seq of murine aortic macrophages defined three clusters of macrophages: ‘Res-like’, which were present in healthy and atherosclerotic aortas, as well as inflammatory and *Trem2*^high^ (triggering receptor expressed on myeloid cells 2) macrophages, which were only present in atherosclerosis.^62^ Whilst inflammatory and Res-like macrophages overlapped significantly with the transcriptomes of *in vitro*-derived M1 and M2 macrophages, respectively, inflammatory macrophages were enriched for *Nrf2* (nuclear factor erythroid2 related factor 2), an anti-inflammatory transcription factor, and *Mrc1* (mannose receptor C Type 1), which encodes CD206, a cell surface marker often used to define M2 macrophages. The newly described *Trem2*^high^ subset did not align to either the M1 or M2 profile.

Interestingly, in contrast to the diversity of macrophage populations, application of scRNA-seq to circulating monocytes in mice suggests that these cells do align well to the classical and non-classical monocyte paradigm.^63^ Ly-6C^high^ and Ly-6C^low^ cells have distinct and well-conserved transcriptomes. The authors of this particular study also address the transcriptional networks of a third population of monocytes. This third population, defined by intermediate expression of Ly-6C in mice and CD16 in humans, has long been recognized and is hypothesized to represent an intermediate developmental stage. The authors demonstrated heterogeneity in the transcriptional profiles of these Ly-6C^int^ cells, and argued that this lent further support to the hypothesis that these cells represent an intermediate developmental stage between classical and non-classical monocytes.

The resolution of scRNA-seq in distinguishing monocyte and macrophage populations is limited by significant overlap in transcriptional profiles between clusters, given their common lineage. Mass cytometry addresses this issue by using a large panel of heavy metal-conjugated antibodies targeting known or suspected differentially expressed proteins.^64^ Application of mass cytometry to the analysis of aortic macrophages described above was able to further subdivide phenotypes that had already been defined by scRNA-seq.^65^ This method of immunophenotyping allows simultaneous analysis of up to 40 proteins and more specific resolution of subsets compared to traditional flow cytometry. Comparison of populations defined using mass and flow cytometry has demonstrated a tendency to misclassify monocytes and macrophages and may account for some of the observed inter-study variation.^14^^,^^35^^,^^66^ Given the complex networks regulating gene expression at the translational and post-translational level, the combination of strategies to interrogate both RNA expression and protein expression is important to appreciate the relevant effector pathways in these cells.

Whilst mass cytometry is, to our knowledge, yet to be applied to cardiac macrophages, emerging data from scRNA-seq and novel experimental models of AMI illustrate macrophage diversity during homoeostasis and following AMI, novel mechanisms by which macrophage populations develop following AMI, and potential mechanism by which they modulate ventricular remodelling.

### 4.2 Cardiac mononuclear cells demonstrate diverse gene expression during homoeostasis

Tissue resident macrophages (TRM) are present in the myocardium during homoeostasis, comprising 7–8% of non-cardiomyocytes in the steady state.^32^ Initial fate mapping experiments in mice divided these cells according to CCR2 expression, demonstrating that CCR2^+^ cells are derived from and dependent on circulating CCR2^+^ monocytes, whereas CCR2^neg^ macrophages are of mixed ontogeny, arising both from the embryonic yolk sac and from foetal liver haematopoietic stem cells. ^67^ This is in contrast to other organs, such as the brain, where TRM are entirely independent of circulating monocytes. These findings have been recapitulated in human hearts, where CCR2^neg^ tissue resident populations have also been demonstrated to be maintained through proliferation, independent of circulating monocytes, whilst CCR2^+^ macrophages depend on circulating CCR2^+^ monocyte recruitment and proliferation.^68^

Use of scRNA-seq has demonstrated greater heterogeneity of myocardial TRMs than previously appreciated, identifying 11 clusters of mononuclear phagocytes with distinct gene expression profiles, comprising one monocyte cluster, four macrophage clusters, three dendritic cells clusters, one myeloid antigen presenting cell cluster, and two clusters of proliferating cells.^69^ Pathway analysis of these subsets suggests differing functions during homoeostasis. The first cluster is enriched for genes involved in reparative functions, such as efferocytosis and angiogenesis, including *Timd4* (T-cell immunoglobulin and mucin containing domain-4), *Lyve1* (lymphatic vessel endothelial hyaluronan receptor 1), and *Igf1* (insulin-like growth factor 1). These cells are entirely maintained through local proliferation, and likely form a subset of the CCR2^neg^ cells described before. A second cluster was unique in its expression of *Ccr2*, and was found to be enriched for genes involved in cell migration, hypoxic response, and the respiratory burst, suggesting a pro-inflammatory role. As previously described, these cells were entirely replaced by differentiation of circulating monocytes. The final populations were of mixed lineage, maintained both by local proliferation and by differentiation of circulating monocytes, and were characterized by enrichment of genes involved in antigen presentation and interferon-stimulated genes, respectively. This study demonstrates the utility of high throughput techniques in both resolving populations that are otherwise described homogenously and identifying novel target pathways.

In addition to these myocardial macrophages, the pericardial cavity, a serous cavity surrounding the heart, has long been known to contain macrophages.^70^^,^^71^ Whilst high throughput methods are yet to be applied to these macrophages, immunophenotyping by flow cytometry groups three distinct myeloid clusters, MHC II^+^ macrophages, GATA6^*+*^ macrophages, and dendritic cells, during homoeostasis.^72^ Bulk RNA-seq demonstrates that the transcriptional profile of GATA6^*+*^ pericardial cavity macrophages is more similar to that of other serous cavity macrophages, namely pleural and peritoneal, than to cardiac TRM, suggesting conserved functions for serous cavity macrophages across different organ systems. Pericardial macrophages were enriched for genes associated with homoeostasis and metabolism, whilst cardiac TRM were relatively enriched for genes involves in inflammation and, whilst this study did not take into account the newly recognized heterogeneity of cardiac TRM, it does illustrate the existence of a distinct populations of macrophages, whose contributions to cardiac homoeostasis and following injury has hitherto not been appreciated.

### 4.3 The heterogeneity and plasticity of macrophage transcriptomes following AMI

Comparison of macrophage populations before and after AMI offers insights into the mechanisms of macrophage differentiation and suggests candidate therapeutic targets to mitigate ventricular remodelling. scRNA-seq of mononuclear phagocytes in the infarcted myocardium 11 days after coronary artery ligation showed preservation of the four macrophage clusters described during homoeostasis, in addition to four novel macrophage populations.^69^ Pathways enriched in the AMI specific populations were involved in the inflammatory response, including TNF production, cell migration and adhesion, and hypoxic signalling.

Analysis of the preserved homoeostatic populations between non-infarcted and infarcted tissue, showed near identical gene expression, with the exception of a number of core TRM genes, including *Timd4, Lyve1*, and *Igf1,* which play important roles in angiogenesis and efferocytosis.^69^ It is well-established that TRM in the infarct zone undergo rapid cell death following AMI and that macrophages at the infarct site are derived from a rapid influx of circulating monocytes.^32^^,^^38^ These data, therefore, illustrate both the remarkable plasticity of circulating monocytes to differentiate into diverse macrophage populations, as well as a core signature of TRM genes involved in reparative functions that are lost following AMI and that may represent novel therapeutic targets.

Whilst TRM are depleted in the infarct zone following AMI, emerging data suggest these cells play critical roles in remodelling following AMI.^73^ Global depletion of TRM, by transient inducible knockout of Cx3cr1, results in increased recruitment of monocytes and macrophages to the remote myocardium and greater ventricular remodelling, with reduced LVEF, increased fibrosis and excess mortality 35 days following coronary artery ligation.^69^ However, selective depletion of *Ccr2^+^* TRM results in reduced monocyte recruitment to the infarcted myocardium and improved ventricular size and function compared to wild types 28 days after an ischaemia–reperfusion (I/R) model of AMI, whilst selective depletion of CD169^+^ TRM resulted in worse ventricular outcomes, with reduced ejection fraction and end-diastolic volume, suggesting both critical and subset-specific roles for TRM populations in ventricular remodelling post-AMI.

**Figure 4:**
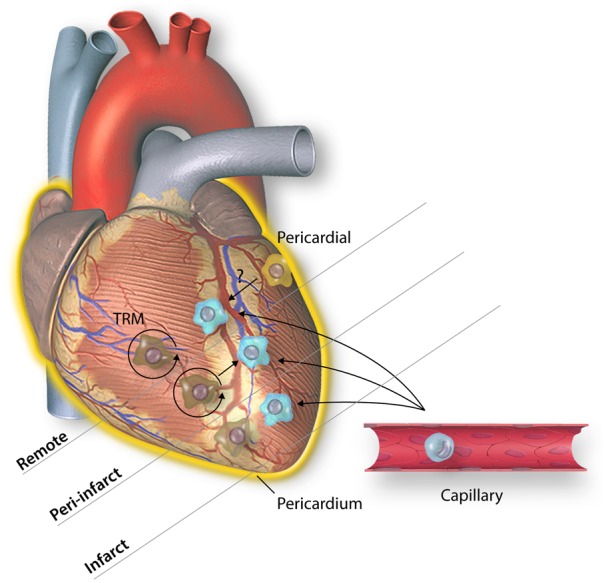
Novel macrophage populations appear to play spatially restricted roles in remodelling of the myocardium following AMI. Following AMI, at the infarct site, TRM undergo rapid cell death, and monocytes rapidly extravasate and differentiate into macrophages. In contrast, in the peri-infarct zone a subpopulation of TRM persist and rapidly proliferate following AMI, whilst monocyte-derived macrophages also contribute to rapid expansion of macrophage numbers at this site. Finally, in the remote myocardium, TRM persist but do not appear to contribute to the increased absolute numbers of macrophages, which is due to infiltration of circulating monocytes. A final, pericardial macrophage population has been identified following AMI. These cells do invade the myocardium but also attach to the epicardial surface and may mediate ventricular remodelling via paracrine signalling.

To understand how TRM may be acting, it is important to consider the roles of macrophages in different regions of the myocardium following injury (*Figure 4*). Whilst TRM are rapidly depleted in the ischaemic zones following AMI, there is rapid expansion of *Lyve1^neg^* TRM specifically in the peri-infarct zone from Day 2 post-coronary artery ligation.^69^ Most studies investigating macrophages after AMI do not differentiate between regions (infarct, peri-infarct, and remote) of the myocardium, and those that do rarely investigate the peri-infarct or remote myocardium specifically, focusing instead on the infarct zone. In the remote myocardium, absolute numbers of macrophages in the remote myocardium expand and persist in the remote myocardium 8 weeks following AMI, and recent analyses suggests it is increased monocyte recruitment and differentiation, rather than TRM expansion, that accounts for this.^15^^,^^69^ Recognizing that macrophage populations may have different roles depending on their location following AMI is an important conceptual shift that may facilitate greater understanding of the mechanisms by which they contribute to ventricular remodelling.

In addition to spatially restricted roles, there appears to be significant temporal variation in macrophage phenotype following AMI. Whilst scRNA-seq is yet to be applied to investigate temporal variation following AMI, bulk RNA-seq of cardiac macrophages illustrates a temporal shift in macrophage phenotype from pro-inflammatory to reparative to resident like.^74^^,^^75^ Whilst macrophages on Day 3 and Day 7 demonstrated predominantly pro-inflammatory and reparative gene expression profiles respectively, both populations showed mixed expression of canonical M1 and M2 genes, again supporting a more nuanced approach to description of M1-M2 phenotypes.^75^

Combination of scRNA-seq to identify candidate pathways and use of transient inhibitors offers a strategy to delineate temporally defined pathogenic pathways. For example, analysis of single-cell transcripts in cardiac macrophages on Day 4 following coronary artery ligation demonstrated the presence of an interferon inducible cell cluster, that was absent in *Irf3^neg/neg^*mice.^76^ Such a cluster has also been identified during homoeostasis and following I/R injury, and, crucially, is distinct to the CCR2^+^ cluster, two populations that previously may have been described synonymously as M1 macrophages.^69^ To investigate the functional relevance of one such novel population, the investigators targeted one candidate pathway. Macrophages of this cluster were enriched for components of the IRF3-IFN (interferon regulatory transcription factor 3–interferon) pathway, which detects cytosolic DNA and initiates an interferon response. Deletion, using constitutive knockout models, of various components of the pathway protected against ventricular remodelling following coronary artery ligation. Crucially, of potential translational application, administration of IFNAR (interferon alpha receptor) neutralising antibody at 12 and 48 h post-AMI abrogated downstream gene expression, improved ventricular size, contractile function, and mouse survival.

The use of a technique to transiently block pathways allows better resolution of the role of particular macrophage phenotypes at different time points following AMI. For example, one might hypothesize that a pathway involved in stimulating fibroblast activity may be beneficial early following AMI but contribute to remodelling if persistently active days to weeks later. Constitutive knockouts are unable to delineate the relative significance of these pathways, whereas techniques to transiently target a pathway would offer translational potential by testing candidate therapies and demonstrating the ideal time points for intervention.

### 4.4 The impact of model systems on macrophage phenotype

The impact of both timing and location on macrophage phenotype suggests significant interplay between the microenvironment and macrophage plasticity. Permanent coronary artery ligation is widely used to model AMI because it causes significant necrosis and a reproducible inflammatory response. However, in the era of widespread clinical access to reperfusion therapy, important differences between permanent and transient coronary artery ligation models should be considered, including the infarct burden and contribution of reperfusion injury. For example, macrophage populations after I/R have a temporally shifted immune response, with macrophage populations at the infarct site peaking 4 days earlier than in the permanent ligation model and having almost completely resolved by 7 days.^77^ The study did not address changes in monocyte or macrophage phenotype, but this is an important outstanding research question. Without confirming the impact of therapeutic targets on ventricular remodelling using an I/R model, candidate studies risk identifying genes and pathways that are of limited translational relevance.

Moreover, recent insights into the role of serous cavity macrophages following visceral injury suggests a fundamental limitation of conventional coronary artery ligation and I/R models, as they require disruption of the parietal pericardium to access the coronary arteries and therefore may interfere with the response of pericardial macrophages to cardiac injury. GATA6^*+*^ serous cavity macrophages are found in the pericardial, pleural and peritoneal cavities during homoeostasis, and their function following injury is best characterized in the peritoneum, an analogous anatomical structure of the abdomen.^78^^,^^79^ These cells are rapidly recruited to the site of acute liver injury, via a non-vascular route that significantly outpaces extravasation of monocytes and contributes to tissue repair through disassembly of necrotic nuclei and promotion of angiogenesis.^80^ Modelling MI using a modified coronary artery ligation model, that leaves the parietal pericardium intact, produces a similar infarct size to conventional coronary artery ligation models but, crucially, mice with an intact pericardium show relatively preserved LV function at 4 weeks, suggesting a role for the pericardium in protecting against ischaemic remodelling.^72^ This cardio-protective effect of the pericardium was abrogated in mice lacking *Gata6^+^*pericardial macrophages. Specifically, mice lacking *Gata6^+^*macrophages showed increased fibrosis of the remote myocardium, impaired LV function and increased LV stiffness at 4 weeks following AMI, modelled using modified coronary artery ligation, despite similar initial infarct sizes, compared to wild-type controls. These cells do migrate into the myocardium but are localized predominantly around the epicardial surface, leading authors to postulate a possible paracrine mechanism of action. Accordingly, future strategies targeting macrophages may need to address not only cells present in the myocardium but also cells attached to the epicardial surface or in the pericardial space, which may contribute to modulation of remodelling via paracrine mechanisms.

## 5. Conclusions and future directions

Given the growing body of evidence demonstrating wide phenotypic variation of macrophages *in vivo* following AMI, understanding their contribution to pathological remodelling requires evolution from the established M1-M2 paradigm. This model assumes a continuum from M1 to M2, whereas *in vivo* evidence suggests a network of diverse macrophage behaviours, driven by a complex network of stimuli.

Interpretation of studies investigating monocyte and macrophage populations is limited by poor phenotypic resolution, and this may account for apparent inconsistencies in the literature. The emergence of unbiased, high throughput single-cell techniques will facilitate more specific identification of candidate macrophage populations and novel signalling pathways for therapeutic application. Furthermore, accounting for temporal and spatial variation will further contribute to our understanding of the regulation of monocyte and macrophage phenotype. These novel techniques will need to be complemented by mechanistic studies to demonstrate the functional significance of newly described phenotypes.

Despite compelling evidence for a key role for monocytes and macrophages in ventricular remodelling following AMI, targeted immunotherapeutic strategies are slow to emerge. Insights into macrophage biology in the context of atherosclerosis led to the first successful clinical trial of immunotherapy in cardiovascular disease (CANTOS) and this success provides a proof of concept for the utility of immunotherapy in this field. In light of the heterogeneity and plasticity of monocytes and macrophages in the heart following AMI, a conceptual shift from targeting arbitrarily and inconsistently defined myeloid cell populations to targeting candidate pathways identified in these cells may bridge the current gap between our current knowledge and therapeutic strategies.

**Conflict of interest:** none declared.

## Funding

This work was supported by the King’s British Heart Foundation Centre for Excellence Award [RE/18/2/34213]. D.B. is supported by an Academy of Medical Sciences Starter Grant for Clinical Lecturers [SGL020\1087].
